# Role of peptidoglycan recycling enzymes AmpD and AnmK in *Acinetobacter baumannii* virulence features

**DOI:** 10.3389/fcimb.2022.1064053

**Published:** 2023-01-13

**Authors:** Ana Tajuelo, María C. Terrón, Mireia López-Siles, Michael J. McConnell

**Affiliations:** ^1^ Intrahospital Infections Laboratory, Instituto de Salud Carlos III (ISCIII), National Centre for Microbiology, Madrid, Spain; ^2^ Universidad Nacional de Educación a Distancia (UNED), Madrid, Spain; ^3^ Electron Microscopy Unit, Scientific-Technical Central Units, Instituto de Salud Carlos III (ISCIII), Madrid, Spain; ^4^ Serra Húnter Fellow, Microbiology of Intestinal Diseases, Biology Department, Universitat de Girona, Girona, Spain

**Keywords:** *Acinetobacter baumannii*, peptidoglycan recycling, biofilm formation, twitching motility, disinfectants, fosfomycin resistance

## Abstract

*Acinetobacter baumannii* is an important causative agent of hospital acquired infections. In addition to acquired resistance to many currently-available antibiotics, it is intrinsically resistant to fosfomycin. It has previously been shown that AmpD and AnmK contribute to intrinsic fosfomycin resistance in *A. baumannii* due to their involvement in the peptidoglycan recycling pathway. However, the role that these two enzymes play in the fitness and virulence of *A. baumannii* has not been studied. The aim of this study was to characterize several virulence-related phenotypic traits in *A. baumannii* mutants lacking AmpD and AnmK. Specifically, cell morphology, peptidoglycan thickness, membrane permeability, growth under iron-limiting conditions, fitness, resistance to disinfectants and antimicrobial agents, twitching motility and biofilm formation of the mutant strains *A. baumannii* ATCC 17978 Δ*ampD*::Kan and Δ*anmK*::Kan were compared to the wild type strain. Our results demonstrate that bacterial growth and fitness of both mutants were compromised, especially in the Δ*ampD*::Kan mutant. In addition, biofilm formation was decreased by up to 69%, whereas twitching movement was reduced by about 80% in both mutants. These results demonstrate that, in addition to increased susceptibility to fosfomycin, alteration of the peptidoglycan recycling pathway affects multiple aspects related to virulence. Inhibition of these enzymes could be explored as a strategy to develop novel treatments for *A. baumannii* in the future. Furthermore, this study establishes a link between intrinsic fosfomycin resistance mechanisms and bacterial fitness and virulence traits.

## 1 Introduction

*Acinetobacter baumannii* (*A. baumannii*) is a Gram-negative pathogen that causes different types of nosocomial infections. Most commonly, it can cause central line-associated bloodstream infections or ventilator-associated pneumonia, but it is also responsible for infections in soft tissues, the skin, and the urinary tract (Lee et al., 2017; Harding et al., 2018). *A. baumannii* is intrinsically resistant to several antimicrobial agents, and since the late 20^th^ century increasing acquired resistance to other antibiotics has been reported (Rolain et al., 2013). Therefore, the emergence of multidrug-resistant (MDR) strains of *A. baumannii* is now recognized as a major global health problem due to the limited options for antibiotic therapy, prompting the World Health Organization (WHO) to declare *A. baumannii* a pathogen of critical priority for which new antimicrobials are urgently needed (Jiang et al., 2021). This can include both discovery of novel antibiotics or potentiating the activity of those currently in use.

Fosfomycin is a broad spectrum antibiotic widely used in clinical practice for treating a range of infections, such as meningitis, otitis, cystitis, respiratory infections, endocarditis or bacteremia (Candel et al., 2019). This is in part due to its high penetration, which allows efficient distribution into many tissues (Sastry and Doi, 2016; Múñez Rubio et al., 2019). In addition, fosfomycin has a favorable safety profile (Iarikov et al., 2015), and lower toxicity compared to other antibiotics such as colistin, whose use has increased over the past years due to its activity against many multidrug resistant bacteria (Spapen et al., 2011; Karaiskos and Giamarellou, 2014; Carretero-Ledesma et al., 2018). Furthermore, fosfomycin has been shown to reduce toxicity caused by nephrotoxic drugs (Karaiskos and Giamarellou, 2014; Sastry and Doi, 2016). However, intrinsic resistance to this antibiotic in *A. baumannii* has hampered its use in treating infections caused by this pathogen (Doi, 2019; Aghamali et al., 2019).

Fosfomycin acts by inhibiting the UDP-*N*-acetylglucosamine enolpyruvyl transferase (MurA). This enzyme is responsible to catalyze the reaction between UDP-*N*-acetylglucosamine (UDP-GlcNAc) with phosphoenolpyruvate (PEP) to form UDP-GlcNAc-enoyl pyruvate plus inorganic phosphate, which is one of the first steps in peptidoglycan synthesis (Silver, 2017). This antibiotic competes with PEP to bind covalently to the enzyme, acting as a PEP analog, which finally results in bacterial cell lysis and death (Silver, 2017; Doi, 2019; Aghamali et al., 2019). It is known that functional MurA is present in *A. baumannii* (Sonkar et al., 2017). In addition, mechanisms associated with fosfomycin resistance in other Gram-negative bacteria, such as the presence of a *fosA* homolog (encoding a glutathione S-transferase that conjugates glutathione to fosfomycin for its inactivation) or changes in the drug transporters GlpT and UhpT have not been described in *A. baumannii* (Gil-Marqués et al., 2018; Aghamali et al., 2019). In contrast, in *A. baumannii* a salvage pathway within the peptidoglycan recycling system has been reported. Specifically, homologs for some of the enzymes involved in the bypass of the enzymatic step catalyzed by MurA have been found in this species (Gil-Marqués et al., 2018). This pathway, present in many Gram-negative species, has been demonstrated to be responsible for resistance to fosfomycin in *Pseudomonas putida* (Gisin et al., 2013), which supports this as the most plausible mechanism resulting in intrinsic resistance to fosfomycin in *A. baumannii*.

In a previous study by our group (Gil-Marqués et al., 2018), we demonstrated that knockout strains of *A. baumannii* lacking *N*-acetyl-anhydromuramyl-L-alanine amidase (AmpD) and anhydro-*N*-acetylmuramic acid kinase (AnmK) enzymes, both acting in the initial steps of the peptidoglycan recycling salvage pathway, featured increased susceptibility to fosfomycin. However, how these mutations affect other pathogenic characteristics of this species has not been studied. Peptidoglycan is an essential component of the bacteria cell wall (Vollmer et al., 2008), so the disruption of its recycling pathway in *A. baumannii* could affect the fitness and virulence of this bacteria. *A. baumannii* presents different virulence factors that contribute to produce the infection in the host, including adherence and biofilm formation that confers to it the ability to survive in the environment and also host cells, surface motility that contributes to stablish the infection, acquisition systems for essential nutrients such as iron or stress resistance (McConnell et al., 2013; Lin and Lan, 2014; Harding et al., 2018). In this context, the aim of this study was to evaluate the role of these two enzymes in some of these traits related to virulence and fitness of *A. baumannii*.

## 2 Materials and methods

### 2.1 Bacterial strains

All bacterial strains used in this study and the assay in which they were used are listed in **Table 1**. Mutant strains and their complemented counterparts were obtained in a previous study by our group (Gil-Marqués et al., 2018). Briefly, the Δ*ampD*::Kan and Δ*anmK*::Kan mutants were constructed in the *A. baumannii* ATCC 17978 strain replacing the wild type genes with a kanamycin resistance cassette through homologous recombination. The pUCp24 plasmid (gentamicin resistance) was used to complement *ampD* and *anmK* mutant strains.

**Table 1:** *Acinetobacter baumannii* (*A. baumannii*) strains used and experiments in which they were engaged.

Strain	Assay*	Reference
*A. baumannii* ATCC 17978	BF, CI, CP, GC, MIC, TEM, TW	ATCC, USA
*A. baumannii* ATCC 19606^T^	TW	ATCC, USA
*A. baumannii* Δ*ampD*::Kan	BF, CI, CP, GC, MIC, TEM, TW	(Gil-Marqués et al., 2018)
*A. baumannii* Δ*ampD*::Kan/pUCp24-*ampD*	BF, CI, CP, GC, MIC, TEM, TW	(Gil-Marqués et al., 2018)
*A. baumannii* Δ*ampD*::Kan/pUCp24	BF, GC, MIC, TW	(Gil-Marqués et al., 2018)
*A. baumannii* Δ*anmK*::Kan	BF, CI, GC, MIC, TEM, TW	(Gil-Marqués et al., 2018)
*A. baumannii* Δ*anmK*::Kan/pUCp24-*anmK*	BF, CI, CP, GC, MIC, TEM, TW	(Gil-Marqués et al., 2018)
*A. baumannii* Δ*anmK*::Kan/pUCp24	BF, GC, MIC, TW	(Gil-Marqués et al., 2018)

*BF, biofilm; CI, Competition index; CP, Cell permeability; GC, Growth curves; MIC, Minimum inhibitory concentration; TW, Twitching; TEM, Transmission electron microscopy.

*A. baumannii* strains were routinely cultured in Mueller Hinton broth (MHB), supplemented, if required, with 10 μg/ml kanamycin (mutant strains) or with 10 μg/ml kanamycin plus 10 μg/ml gentamicin (complemented mutant strains). For long-term storage, strains were kept in Luria Bertani (LB) media containing 20% glycerol (v/v) and stored at -80 °C. Bacteria were freshly plated from stocks for each experiment.

### 2.2 Transmission electron microscopy

For TEM ultrastructural analysis, pellets of the *A. baumannii* ATCC 17978 wild type strain, mutants and complemented strains harvested during exponential growth (OD_600_ = 0.5) were chemically fixed in 0.1 M Na_2_HPO_4_ buffer pH 7.4, 3% glutaraldehyde and 4% paraformaldehyde for 150 min at 4 °C. Cells were centrifuged and washed in Na_2_HPO_4_ buffer three times. Postfixation was performed with a mixture of 1% osmium tetroxide and 1.5% potassium ferrocyanide for 1.75 h at 4 °C. Subsequent treatments consisted of 0.15% tannic acid for 1 min at room temperature and 2% uranyl acetate for 1 h at room temperature in the dark. Dehydration was carried out in increasing concentrations of ethanol (50, 75, 90, 95, and three times with 100%) for 10 min each at 4 °C. Infiltration was performed at room temperature and agitation, using increasing concentrations of epoxy-resin (25, 50, 75 and 100%). Polymerization was performed at 60 °C for 48 h. Ultrathin sections of the samples (50-70 nm) were obtained with a Leica EM UC6 ultramicrotome and harvested on 100 mesh Formvar coated copper grids. Staining was carried out following standard procedures with saturated uranyl acetate and 2% lead citrate. Images were captured at nominal magnifications of 15,000 × to 67,000 × with a CCD (Charged Coupled Device) FEI Ceta camera on a Tecnai 12 electron microscope (FEI) operated at 120 kV.

For measurement of the cell wall dimensions, bacteria were selected with the outer and inner membrane, and the peptidoglycan layer unequivocally visible to ensure the structures were perpendicular to the surface section. Images were recorded at a nominal magnification of 67,000 ×, corresponding to a pixel size of 0.15 nm. Images were opened in Fiji (Schindelin et al., 2012) software and a line profile was drawn from the innermost part of the inner membrane to the outermost part of the outer membrane, perpendicular to the peptidoglycan layer with the length of the line representing the dimensions of the cell wall as described in Bleck et al. (2010).

### 2.3 Cell permeability assay

To determine membrane permeability of *A. baumannii* strains, SYTOX green (S7020, Thermo Fisher) and 1-N-phenylnaphthylamine (NPN, 104043, Sigma-Aldrich) stains were used. Strains were grown until the exponential phase and adjusted to a final OD_600_ = 0.5 in PBS supplemented with 1 μM SYTOX green. 100 μl of each bacterial suspension were placed into the appropriate well of a black microtiter plate (clear bottom) (353219, Falcon). Fluorescence (λex = 504 nm, λem = 523 nm) was measured using an automated plate reader (M200 Infinite Pro, Tecan). For NPN assay, 150 μl of the bacterial suspensions grown until the exponential phase and adjusted to OD_600_ = 0.5 in 5 mM HEPES (pH = 7.2) were transferred to the wells of a plate as indicated previously. 50 μl of a 40 μM NPN solution in 96% ethanol were added and fluorescence (λex = 350 nm, λem = 420 nm) was measured immediately as indicated above. Permeability in the presence of 5mg/ml of the detergent SDS was carried out as a positive control of compound uptake.

### 2.4 *In vitro* growth curves

Growth in iron-rich media, iron-limiting conditions and serum was tested. *A. baumannii* strains were cultured in 5 ml of MHB overnight at 37 °C and then adjusted as appropriate as previously reported (Gil-Marqués et al., 2018), with slight modifications. Specifically, to elaborate *in vitro* growth curves in MHB (iron-rich condition) or inactivated human serum (SLCC3239, Sigma-Aldrich), 100 μl of bacteria at a concentration of 10^6^ CFU/ml were used. As growth curves were performed without antibiotic pressure, a higher inoculum was used to minimize plasmid loss effect. To monitor growth in iron-limiting conditions, 200 μl of the overnight cultures adjusted to a concentration of 10^5^ CFU/ml in MHB were supplemented with the iron chelator 2, 2’-bipyridyl (Bip) at a final concentration of 150 μM, following a previously reported method (Carretero-Ledesma et al., 2018). All experiments were carried out in 96-well flat bottom polystyrene microplates (351172, Falcon). Growth at 37 °C was assessed by measuring the OD_600_ every 30 min over 24 h using an automated reader (M200 Infinite Pro, Tecan). All assays were performed at least in duplicate.

### 2.5 *In vitro* competition indices

Four different strain combinations were analyzed in separate experiments: *A. baumannii* ATCC 17978 and *A. baumannii* Δ*ampD*::Kan; *A. baumannii* ATCC 17978 and *A. baumannii* Δ*anmK*::Kan; *A. baumannii* ATCC 17978 and *A. baumannii* Δ*ampD*::Kan/pUCp24-*ampD*; *A. baumannii* ATCC 17978 and *A. baumannii* Δ*anmK*::Kan/pUCp24-*anmK*.

*In vitro* competition experiments were carried out using a protocol from a previous study (Carretero-Ledesma et al., 2018). Overnight cultures of bacterial strains were diluted to a final concentration of 10^5^ CFU/ml and mixed in 1:1 ratio in MHB. After 24 h, aliquots from the cultures were plated on MH agar plates and MH plates containing 10 µg/ml of kanamycin to select for *A. baumannii* mutant strains or MH plates containing 10 µg/ml of kanamycin and 10 µg/ml of gentamicin to select for *A. baumannii* complemented mutant strains. Competition indices (CI) were obtained from the following formula:


$$CI=\frac{\frac{CFU_{mut}}{CFU_{wt}}}{\frac{CFU_{mut0}}{CFU_{wt0}}},$$

where the number of CFU recovered from the mutant strain (CFU_mut_) with respect to the number of CFU recovered from the wild type *A. baumannii* ATCC 17978 strain (CFU_wt_), is divided by the number of CFU in the mutant inoculum (CFU_mut0_) with respect to the number of CFU in the wild type inoculum (CFU_wt0_). CI < 1 represents an increased growth of the wild type strain, C = 1 a similar growth of both strains and CI > 1 an increased growth of the mutant strain. All assays were performed in triplicate.

### 2.6 Susceptibility to disinfectants and other antimicrobial agents

The broth microdilution method was used to determine the minimum inhibitory concentration (MIC) values for disinfectants chlorhexidine (282227-1G, Sigma) and ethanol (141086.1212, Panreac), for deoxycholate (30970-25G, Sigma), a secondary bile acid that emulsify fats and alters the permeability of lipid membranes being considered a natural antimicrobial agent (Begley et al., 2005; Urdaneta and Casadesús, 2017), the chelating agent EDTA (A2937, Panreac) and the detergent SDS (A2263, Panreac). MHB II was used according to the Clinical and Laboratory Standards Institute for antimicrobials (CLSI) recommendations (CLSI, 2017). A culture of the corresponding *A. baumannii* strain adjusted to 10^6^ CFU/ml was added to wells of a 96-well U-shaped bottom polystyrene microplate (140935, Biotech) containing two-fold serial dilutions of each disinfectant (0.06 - 0.0001 mM for chlorhexidine, 8.6 - 0.02 mM for ethanol, 120 - 2.4 mM for deoxycholate and 0.014 - 0.000027 mM for EDTA and SDS). Microplates were incubated at 37 °C for 24 h. The MIC was established as the lowest disinfectant concentration at which no growth was observed. MIC analyses were performed in triplicate for each strain.

### 2.7 Biofilm formation

Biofilm formation was determined following previously described protocols (Carretero-Ledesma et al., 2018; Domenech and García, 2020) with some modifications. Overnight cultures of each strain were adjusted to a final concentration of 10^8^ CFU/ml in Mueller Hinton II broth. Each bacterial suspension (200 µl) was added into a well of a U-shaped polystyrene 96-well plate (140935, Biotech) and incubated without shaking at 37 °C for 24 h. After incubation, media was discarded and the adherent cells were washed with PBS. Biofilm staining was performed with a 1% crystal violet solution for 15 min at room temperature. The crystal violet solution in excess was removed, and the plates were washed twice as indicated previously. The stain was eluted from the adherent cells by adding 200 μl of 70% ethanol (v/v) to each well. Then, the absorbance at 595 nm was measured on a microplate reader (Epoch 2, BioTek). Percentages of biofilm formation were calculated by comparing the absorbance of mutant and complemented strains with respect to the absorbance of the wild type strain. The assay was performed six times.

### 2.8 Twitching motility assay

To analyze twitching motility, a previously reported protocol (Carretero-Ledesma et al., 2018) was used with minor modifications. Each strain was grown overnight in MHB. Prior to initiate the twitching motility assay, cultures were adjusted by dilution with PBS to a final concentration of 10^9^ CFU/ml. LB plates containing 0.3% agarose (801000, Pronadisa) were prepared and inoculated the same day with 2 μl of the adjusted bacterial suspension placed in the interphase between the medium and the bottom of the Petri dish. Plates were allowed to dry for 5 min and then incubated at 37 °C with a humidity saturated atmosphere. The diameter of culture surface extension was measured after 32 h of incubation. *A. baumannii* ATCC 19606^T^, a non-motile strain (Eijkelkamp et al., 2011), was used as a negative control. For each isolate, the assay was performed three times on separate days.

### 2.9 Statistical analyses

All analyses and data plotting were performed using SPSS (IBM) and Prism 5 v.5.01 (GraphPad Software). Given the normal distribution of the data, as assessed by Shapiro-Wilks test, parametric statistical tests were used. Growth in different conditions were compared using the Student t-test for pairwise analyses. Competition indices were compared to an expected value of 1 using the Wilcoxon signed rank test and differences between groups were also determined using the Student t-test. Cell permeability, biofilm production, and twitching motility were compared using a one-way ANOVA, and differences between groups were determined using the Tukey post-hoc test. *p*-values < 0.05 were considered statistically significant.

## 3 Results

### 3.1 Effect of *ampD* and *anmK* deletion in cell morphology and cell wall thickness

**Figure 1:**
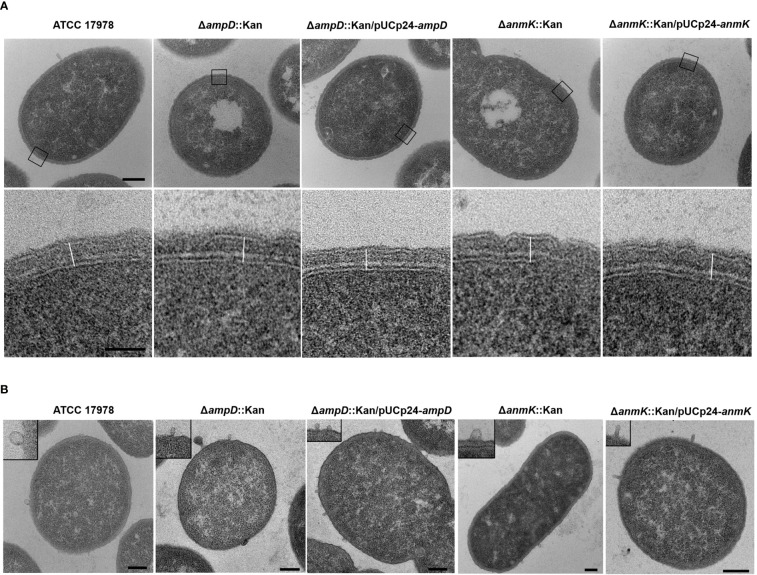
Morphology of the cell wall of *Acinetobacter baumannii* strains by TEM. **(A)** TEM images show an overview of a cell from the wild type strain and the respective mutants or complemented strains (upper row). The box marks areas used for cell wall measurement, which are shown in detail in the lower row. The outermost structure is the outer membrane with a thin electrone dense layer of lipopolysaccharide facing the exterior. The inner membrane separates the cell wall from the cytoplasm. Between outer and inner membrane the peptidoglycan layer is seen as an electron dense layer. The line used for measurements is represented. Scale bars: upper row = 200 nm, lower row = 50 nm. **(B)** TEM images show the presence of outer membrane vesicles (OMV) around a cell of each bacteria strain with the insert in each panel showing a detail of them. Scale bar: 200 nm.

To determine whether *ampD* or *anmK* deletion affects peptidoglycan structure, *A. baumannii* strains were visualized by TEM (**Figure 1A**). All strains presented similar morphology and cell wall thickness (**Table 2**), indicating that the absence of AmpD and AnmK enzymes does not result in gross changes in membrane ultrastructure. Interestingly TEM images revealed the presence of outer membrane vesicles (OMV) in all strains analysed (**Figure 1B**), indicating that *A. baumannii* strains lacking AmpD or AnmK are still able to release OMV.

**Table 2:** Dimensions of the cell wall of *Acinetobacter baumannii* (*A. baumannii*) strains.

Strain	OM-IM*
*A. baumannii* ATCC 17978	32.5 ± 3.9 nm
*A. baumannii* Δ*ampD*::Kan	33.1 ± 2.2 nm
*A. baumannii* Δ*ampD*::Kan/pUCp24-*ampD*	31.8 ± 2.8 nm
*A. baumannii* Δ*anmK*::Kan	31.7 ± 2.4 nm
*A. baumannii* Δ*anmK*::Kan/pUCp24-*anmK*	32.5 ± 2.9 nm

*Measurement of the cell wall from the outer membrane (OM) to the inner membrane (IM). Mean ± standard deviation of at least 20 cells.

### 3.2 Effect of AmpD and AnmK on cell permeability

**Figure 2:**
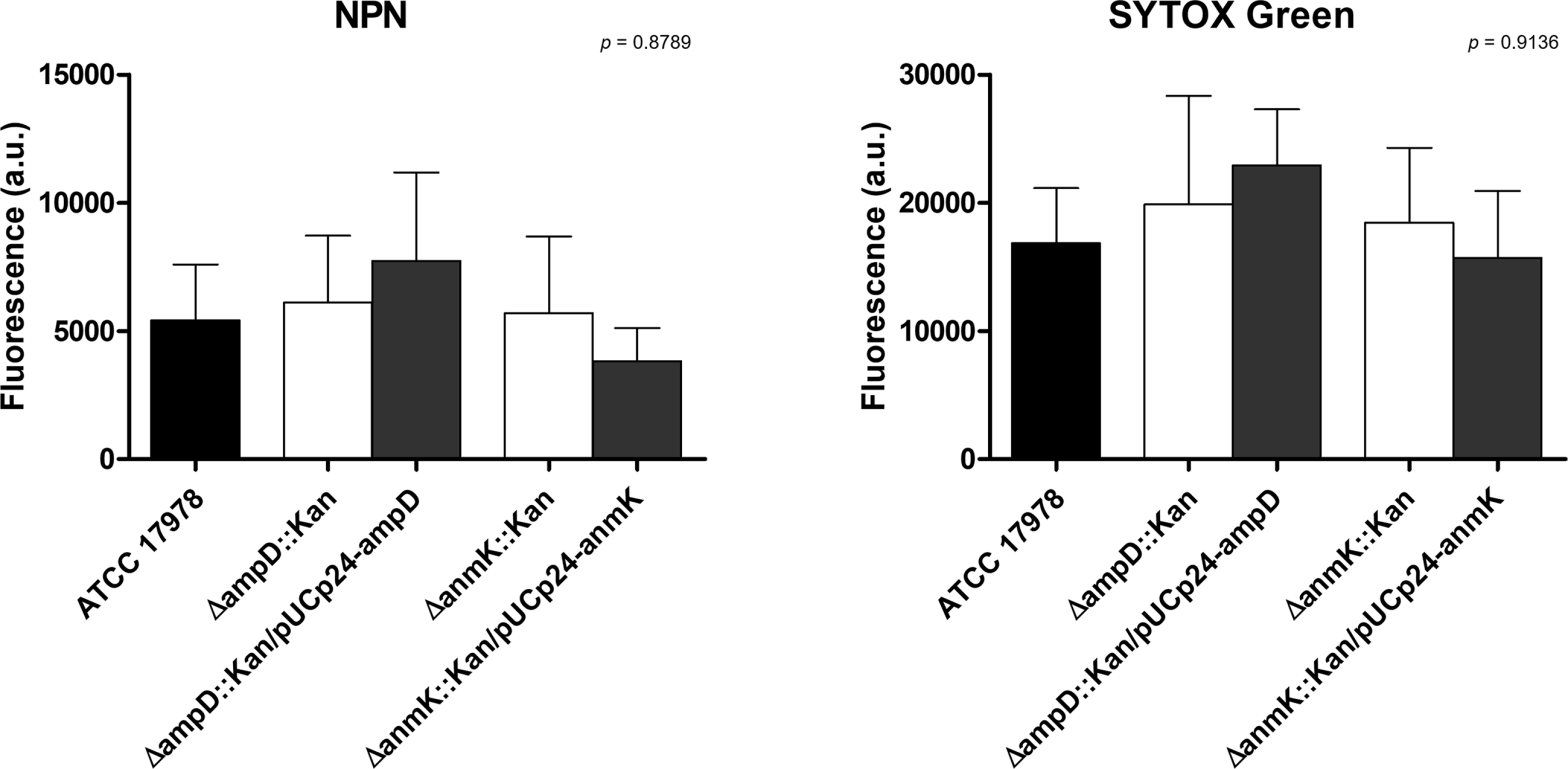
Cell permeability of *Acinetobacter baumannii* strains. Effect of *ampD* and *anmK* deletion on the membrane permeability as measured by the 1-N-phenylnaphthylamine (NPN) (left) and SYTOX Green (right) uptake assays. Bars represent the average of three separate assays, with error bars representing the standard deviation. No significant differences were found between replicates (*p =* 0.8789 and *p* = 0.9136 for NPN and SYTOX Green, respectively), as assessed by ANOVA followed by Tukey’s Multiple Comparison Test.

To evaluate if *ampD* and *anmK* deletion affected membrane permeability of *A. baumannii*, accumulation assays were carried out using the fluorescent stains NPN and SYTOX Green, neutral and positively charged compounds, respectively (**Figure 2**). There were no significant differences in intracellular accumulation of NPN and SYTOX Green in the mutant strains compared to the wild type strain (*p* > 0.05), indicating that the absence of AmpD or AnmK does not result in increased membrane permeability to these compounds. The presence of SDS, that disrupts the cell membrane, resulted in an almost two-fold significant increase in membrane permeability of ATCC 17978 (*p* = 0.038, **Supplementary Figure 1**).

### 3.3 *In vitro* growth of *A. baumannii* strains

**Figure 3:**
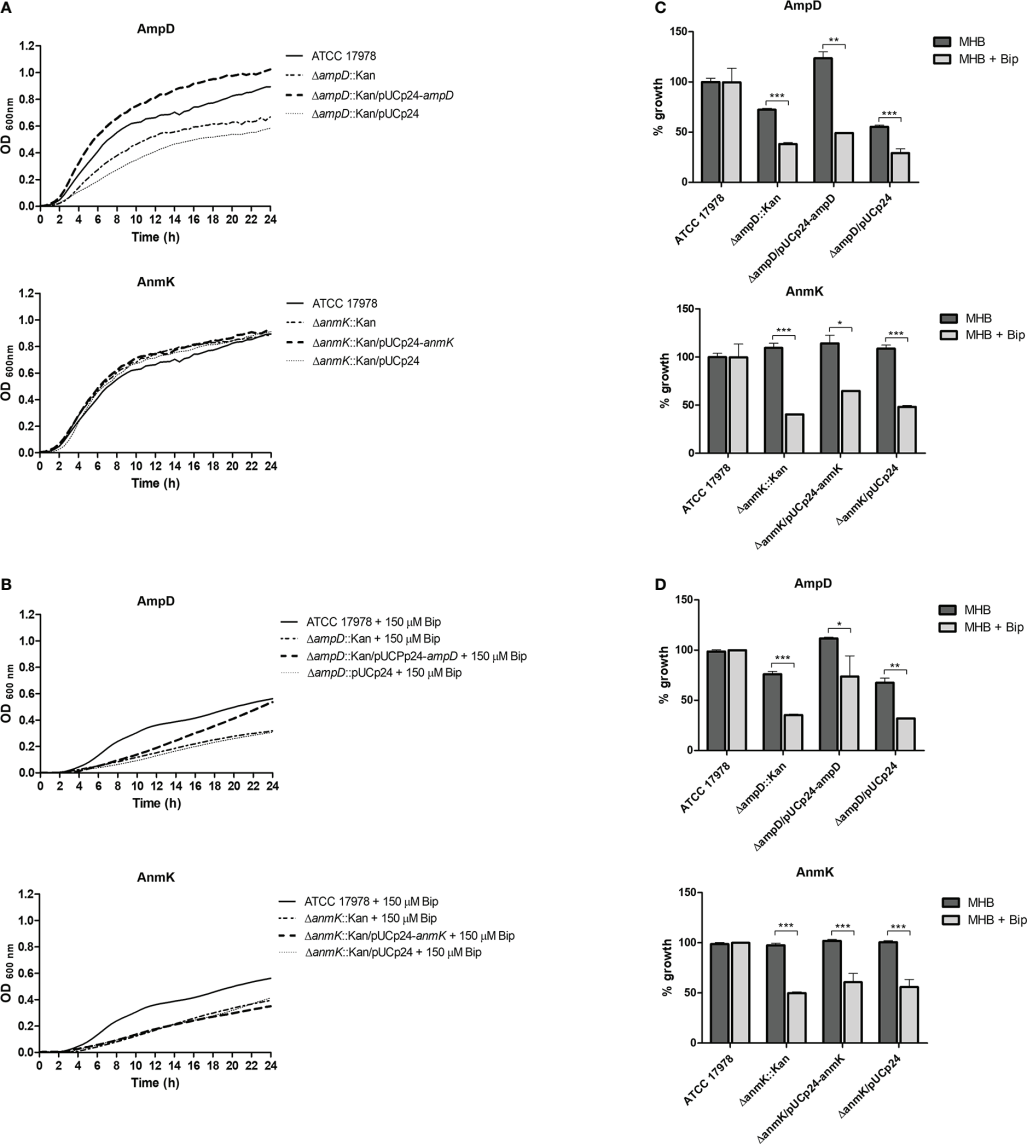
*In vitro* growth of *Acinetobacter baumannii* strains in Mueller Hinton broth (MHB). **(A)** Growth curves over 24 h of *A. baumannii* strains in media MHB. **(B)** Growth curves over 24 h of *A. baumannii* strains in MHB supplemented with 150 μM of the iron chelator 2, 2'-bipyridil (Bip). **(C)** Percentage of growth with respect to the *A. baumannii* ATCC 17978 strain in MHB or MHB + Bip at exponential growth phase (6 h). **(D)** Percentage of growth with respect to the *A. baumannii* ATCC 17978 strain in MHB or MHB + Bip at stationary growth phase (24h). * *p*< 0.05, ** *p*< 0.01 and *** *p*< 0.001, Student’s t-test.

The effect of *ampD* and *anmK* absence on *A. baumannii* growth in rich media (MHB) and in iron limiting conditions was assessed over 24 h (**Figure 3**). The *ampD* deletion resulted in reduced growth compared to the parental strain ATCC 17978 (**Figure 3A**). Complementation of the mutation with a wild type copy of the gene (Δ*ampD*::Kan/pUCp24-*ampD*) completely restored the growth defect observed, whereas the Δ*ampD*::Kan mutant containing an empty plasmid did not restore growth. A different result was obtained with the Δ*anmK*::Kan mutant, which grew similarly to the parental strain, as did the complemented strain and the strain containing an empty plasmid.

Under iron limiting conditions strains lacking AmpD or AnmK both demonstrated reduced growth compared to the wild type parental strain (**Figure 3B**). This difference was observed at both exponential (38 and 40% of growth, respectively) and stationary phase (35 and 50% of growth, respectively) time points (**Figures 3C, D**). Moreover, under this situation complementation with a wild type copy of the genes did not re-establish growth to the parental strain level in either case at exponential phase, showing a similar growth to the strain containing an empty plasmid. In the stationary phase, a partially restored wild type phenotype was only observed when the Δ*ampD*::Kan mutant was complemented, although significant differences compared to wild type strain were maintained. In addition, we assessed growth in human serum, and all strains including ATCC 17978 showed a marked growth defect with an OD_600_< 0.4 after 24 h (**Supplementary Figure 2**).

### 3.4 Effect of *ampD* and *anmK* deletion on fitness of *A. baumannii*

**Figure 4:**
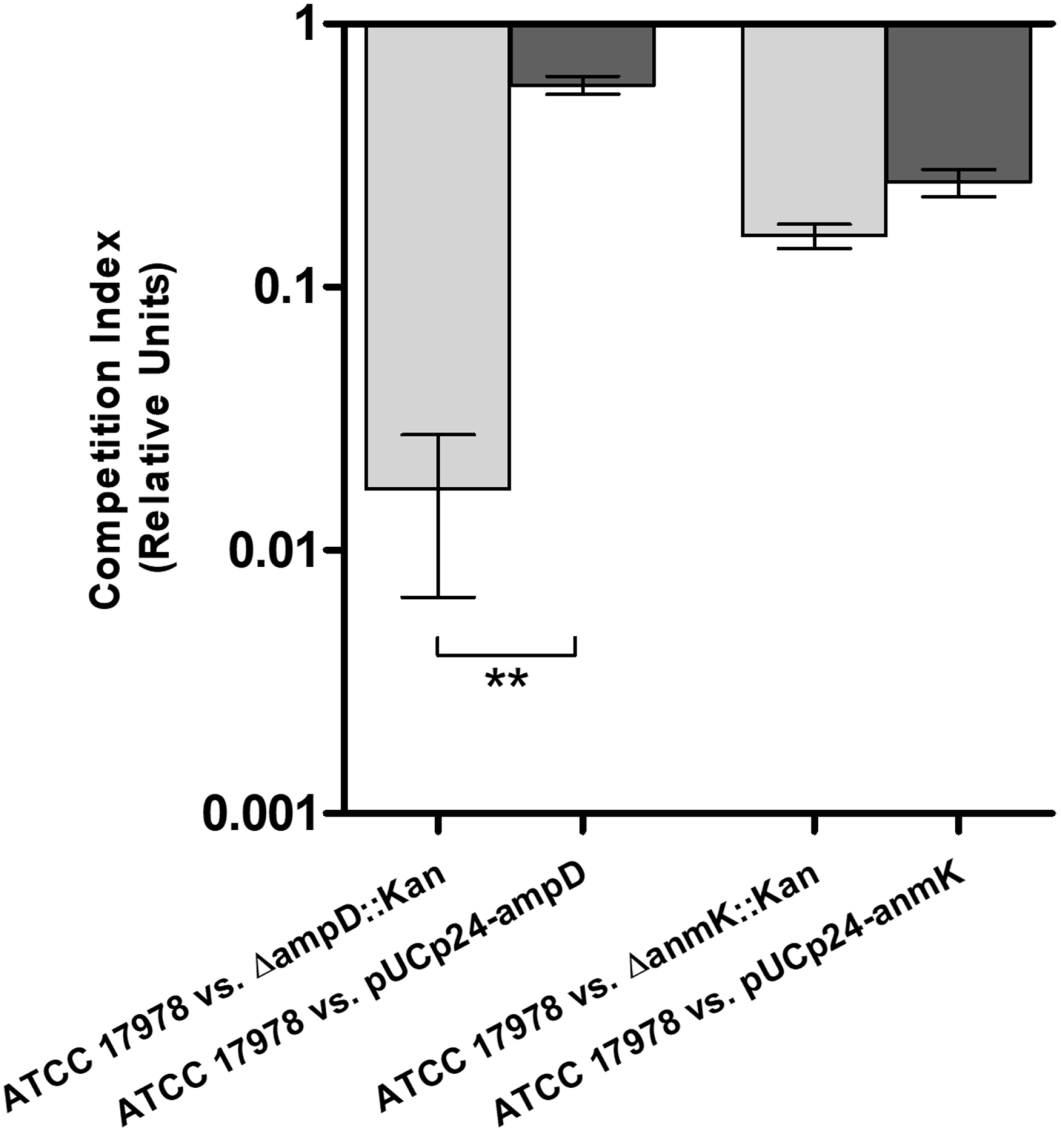
Fitness of mutant and complemented strains of *Acinetobacter baumannii* with its wild type counterpart in Mueller Hinton broth. Competition indices comparing ATCC 17978 and Δ*ampD*::Kan, ATCC 17978 and Δ*ampD*::Kan/pUCp24-*ampD*, ATCC 17978 and Δ*anmK*::Kan, and ATCC 17978 and Δ*anmK*::Kan/pUCp24-*anmK* in MHB. Bars represent the mean ± standard deviation of three independent assays. ** *p* = 0.0042 compared to mutant strain, Student’s t-test.

To further characterize changes in fitness as a consequence of *ampD* and *anmK* deletion, competition indices were determined at 24 h in MHB by comparing the growth of the Δ*ampD*::Kan, Δ*anmK*::Kan and their complemented strains, Δ*ampD*::Kan/pUCp24-*ampD* and Δ*anmK*::Kan/pUCp24-*anmK*, to ATCC 17978 when grown together (**Figure 4**). Despite difference to 1 was not statistically significant in neither case according to the Wilcoxon test due to the low number of replicates, *A. baumannii* Δ*ampD*::Kan demonstrated a marked loss of fitness compared to ATCC 17978 (CI = 0.017), whereas the complemented strain Δ*ampD*::Kan/pUCp24-*ampD* showed a significant restoration of this fitness (CI = 0.59) (*p* = 0.0042). Fitness loss was less reduced in *A. baumannii* Δ*anmK*::Kan, (CI = 0.16) compared to Δ*ampD*::Kan mutant which was partly restored by its complemented counterpart (CI = 0.25).

### 3.5 Effect of AmpD and AnmK on susceptibility to disinfectants and other antimicrobial agents

The MIC for chlorhexidine, ethanol, deoxycholate, EDTA and SDS was determined to assess if deletion of *ampD* or *anmK* genes in *A. baumannii* affected susceptibility to different antimicrobial agents (**Table 3**). The absence of AmpD and AnmK did not affect susceptibility to chlorhexidine, ethanol, EDTA or SDS. In contrast, ATCC 17978 Δ*ampD*::Kan strain was slightly more susceptible to deoxycholate than the wild type strain and complementing the mutation returned susceptibility to wild type levels.

**Table 3:** Minimum inhibitory concentration (MIC) of *Acinetobacter baumannii* (*A. baumannii*) strains to disinfectants and antimicrobial agents.

Strain	Chlorhexidine (mM)	Ethanol (mM)	Deoxycholate* (mM)	EDTA (mM)	SDS (mM)
*A. baumannii* ATCC 17978	0.03	1	> 120	0.007	0.002
*A. baumannii* Δ*ampD*::Kan	0.03	1	120	0.007	0.002
*A. baumannii* Δ*ampD*::Kan/pUCp24-*ampD*	0.03	1	> 120	0.007	0.002
*A. baumannii* Δ*ampD*::Kan/pUCp24	0.03	1	> 120	0.007	0.002
*A. baumannii* Δ*anmK*::Kan	0.03	1	> 120	0.007	0.002
*A. baumannii* Δ*anmK*::Kan/pUCp24-*anmK*	0.03	1	> 120	0.007	0.002
*A. baumannii* Δ*anmK*::Kan/pUCp24	0.03	1	> 120	0.007	0.002

*The exact MIC for deoxycholate could not be established because it was not soluble at concentrations above 120 mM.

### 3.6 Effect of *ampD* and *anmK* deletion on biofilm production

**Figure 5:**
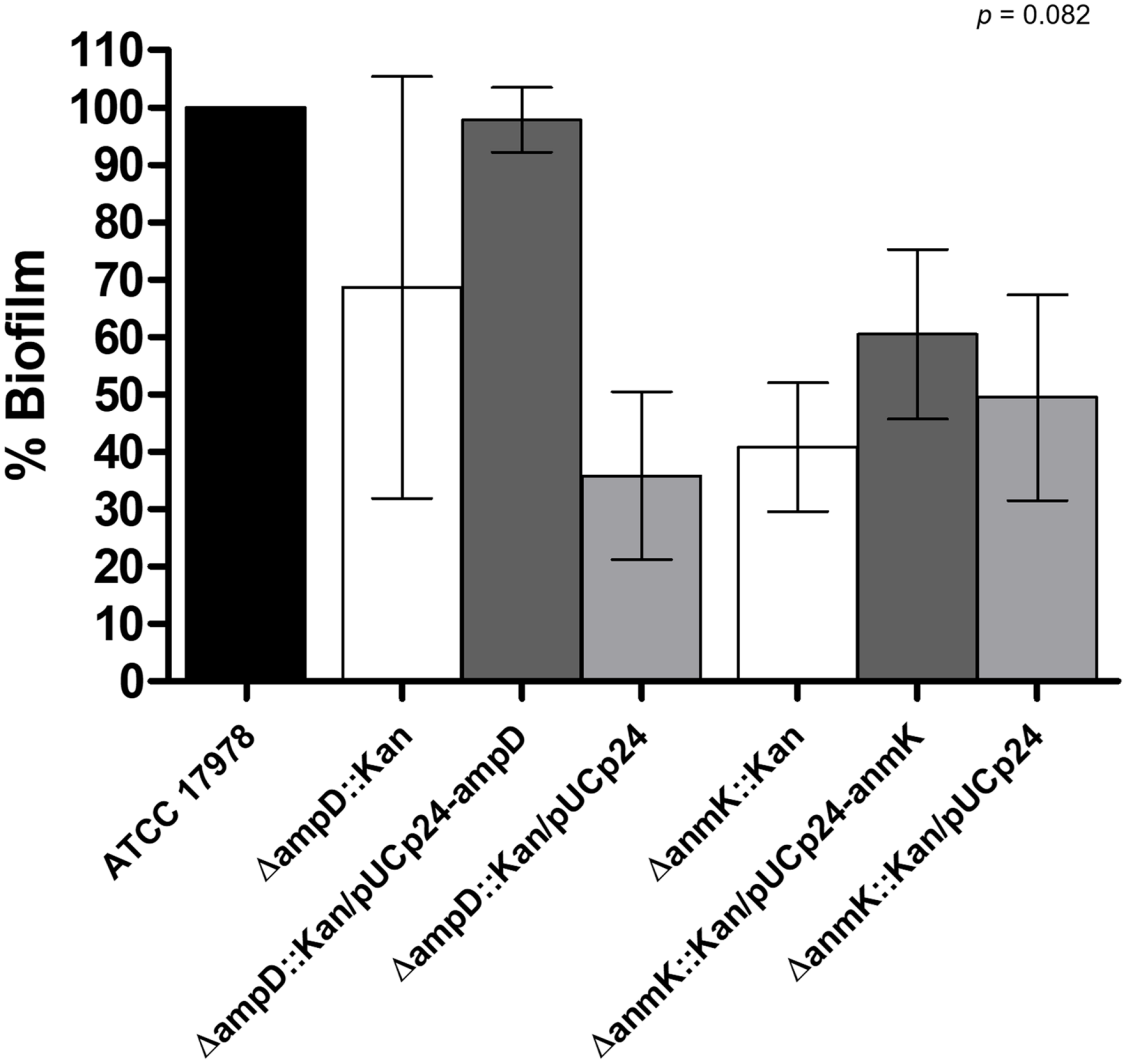
Effect of Δ*ampD* and Δ*anmK* deletion on biofilm production. Percentage of biofilm production was determined for mutants strains (Δ*ampD*::Kan and Δ*anmK*::Kan), their complemented strains (Δ*ampD*::Kan/pUCp24-*ampD* and Δ*anmK*::Kan/pUCp24-*anmK*), and the complemented strains with an empty plasmid (Δ*ampD*::Kan/pUCp24 and Δ*anmK*::Kan/pUCp24) respect to the wild type ATCC 17978 strain. Bars represent the average of six separate assays, with error bars representing the standard deviation. No significant differences were found between replicates (*p =* 0.082), as assessed by ANOVA followed by Tukey’s Multiple Comparison Test.

Biofilm production in the ATCC 17978, Δ*ampD*::Kan and Δ*anmK*::Kan mutants and their complemented counterpart strains was assessed (**Figure 5**). Absence of AmpD reduced the ability to form biofilm to 69% after 24 h compared to the parental strain, while a more marked phenotype was observed for *anmK* deletion, reducing biofilm formation to 41%. Complementation with a wild type copy of the gene only resulted in a restoration of biofilm production in the Δ*ampD*::Kan mutant, whereas complement with an empty plasmid produced similar biofilm to mutant strain, indicating that the decreased biofilm production was due to AmpD absence. These differences were not statistically supported (*p* = 0.082) because of the dispersion of data between the six replicates.

### 3.7 Effect of *ampD* and *anmK* deletion on twitching motility

**Figure 6:**
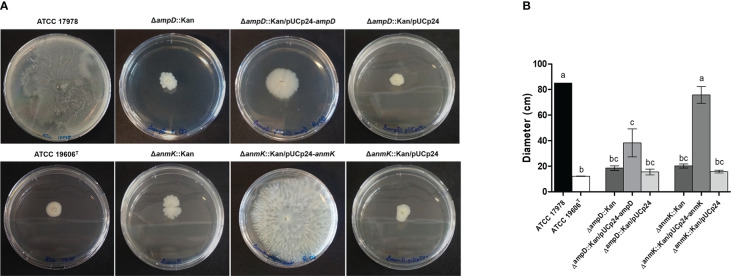
Twitching motility displayed by *Acinetobacter baumannii* strains. **(A)** Surface motility on semisolid media of wild type strain, the respective mutants and complemented strains after 32 h of incubation. **(B)** Measurement of surface extension for each bacterial strain. Error bars represent the standard deviation of triplicate experiments. Different superscript letters indicate significant difference (*p*< 0.05) between the strains as assessed by ANOVA followed by Tukey’s Multiple Comparison Test.

Lastly, to determine if the lack of *ampD* and *anmK* affected *A. baumannii* surface motility, we determined twitching motility, based on the ability of the bacteria to translocate on the surface of a semisolid media over 32 h of incubation (**Figure 6**). Δ*ampD*::Kan and Δ*anmK*::Kan mutants showed an important loss in twitching motility (78 and 76% reduction, respectively) compared to *A. baumannii* ATCC 17978 (*p<* 0.001*).* Complementation of Δ*anmK*::Kan mutant totally restored surface motility to parental level. In contrast, a mild increase in surface motility was observed when complementing Δ*ampD*::Kan mutant. Strains complemented with an empty plasmid displayed the same twitching as the negative control strain and knockout mutants.

## 4 Discussion

*A. baumannii* resistance to fosfomycin has been linked to the presence of a functional peptidoglycan recycling pathway, as its disruption has been shown to increase susceptibility to this antibiotic (Gil-Marqués et al., 2018). However, how mutations in enzymes involved in this pathway affect bacterial physiology and virulence features has not been characterized. In the present study we have explored the role of the peptidoglycan recycling pathway enzymes AmpD and AnmK in multiple virulence associated traits. Since they are involved in an early and late step of the recycling route, respectively, this has allowed us to elucidate mild and more severe effects in virulence traits that result when altering the peptidoglycan recycling pathway at different enzymatic steps.

In addition to maintaining cell shape, the peptidoglycan is responsible for imparting strength and resistance to osmotic pressure (Cava et al., 2011). Therefore, we first explored if mutations in enzymes in the peptidoglycan recycling pathway affected cell morphology. TEM images demonstrate that *A. baumannii* strains lacking AmpD or AnmK showed no differences in morphology or cell wall thickness compared to the wild type strain. This could be because in our experimental setting, *de novo* synthesis of peptidoglycan is not interrupted and the UDP-MurNAc is supplied by this route in mutant strains. In addition, we observed that OMV were produced in both mutants. Production of OMV has been observed in several strains of *A. baumannii* (Kwon et al., 2009; Jin et al., 2011; Rumbo et al., 2011; McConnell et al., 2011). Reduced levels of crosslinks between the peptidoglycan and the outer membrane, which is modulated through the peptidoglycan recycling (Schwechheimer and Kuehn, 2015; Kim et al., 2021), have been shown to influence OMV release. However, our results indicate that altering AmpD and AnmK in peptidoglycan recycling pathway do not eliminate this process, although further analyses to quantify the OMVs produced for these strains will be of interest to compare with the wild type levels.

Deletion of *ampD* and *anmK* did not affect membrane permeability as observed when we analyzed the ability of NPN and SYTOX Green, neutral and positively charged fluorescent stains, respectively, to penetrate into the cell. These findings are in line with those reported in a previous study by our group (Gil-Marqués et al., 2018) in which we observed that there were no differences in permeability to ethidium bromide, another positively charged stain, in the same strains. Bacterial cells normally exclude these stains, which can only penetrate into the cell when membrane damage has occurred. Taken together, the results obtained with the different stains demonstrated that lack of AmpD and AnmK does not significantly alter the permeability of cell membrane with respect to these dyes.

Although no structural differences were observed, we wanted to analyze how bacterial fitness was affected by loss of AmpD and AnmK. Peptidoglycan recycling is not essential for *in vitro* growth, but provides metabolites that can be reused to synthesize more peptidoglycan and also as an energy source (Park and Uehara, 2008; Fisher and Mobashery, 2014). In fact, many bacteria remodel as much as half of their peptidoglycan per generation, and cell wall recycling and synthesis are tightly coordinated to preserve bacterial integrity (Johnson et al., 2013; Dhar et al., 2018). We demonstrate that, under laboratory conditions, loss of AmpD is associated with a more marked reduction in fitness than absence of AnmK, as we observed a marked defect in growth relative to the parental strain, both in iron-rich and iron-limiting conditions. In addition, this was confirmed with the lower competition index obtained for the Δ*ampD*::Kan mutant. Differences in inoculum between growth curves and competition experiments may explain why fitness loss is more evident in the latter. The differences in fitness found in this study between both mutants could be due to the fact that the amidase AmpD participates in an initial step of peptidoglycan recycling and thus its absence results in a more complete blockage of peptidoglycan recycling (Gil-Marqués et al., 2018). In addition, AmpD hydrolyzes anhydromuropeptides in the cytoplasm yielding not only products that are involved in the recycling pathway (anhMurNAc), but also Ala-Glu-DAP, that can be incorporated to *de novo* pathway of peptidoglycan (Gil-Marqués et al., 2018). On the other hand, AnmK is involved in the conversion of anhMurNAc to MurNAc-P. This is an enzymatic step that takes place after the hydrolysis of the anhydromuropeptide by AmpD (Gisin et al., 2013). Thus, lack of AnmK function likely only alters the yield of UDP-MurNAc obtained from anhMurNAc affecting the recycling pathway but not peptidoglycan *de novo* synthesis. Similar results were observed in other species such as *Pseudomonas aeruginosa* which has three closely related AmpD enzymes (Rivera et al., 2016), and the triple knockout mutant presented a marked decrease in fitness (Moya et al., 2008). Reduced fitness has also been observed in a *Salmonella typhimurium ampD* mutant in a murine model of infection (Folkesson et al., 2005). We observed that complementation with a plasmid encoding the deleted genes did not always restore the wild type phenotype in the experiments carried out in this work, especially when complementing *anmK* mutant. Because carrying a plasmid has a fitness cost in the bacterium (San Millan and MacLean, 2017; Alonso-Del Valle et al., 2021), we hypothesize that the bacteria could prefer to maintain fitness rather than express the plasmid gene considering that AnmK plays a less important role in the peptidoglycan integrity maintenance. Lastly, we observed some differences in growth curves compared to previous data (Gil-Marqués et al., 2018) as in the present study more efficient complementation is observed by ectopic expression of AmpD and AnmK. This may be due to the higher inoculum used in the present study to minimizing plasmid loss during growth curve experiments.

In addition, it is interesting to note that peptidoglycan recycling also has a regulatory role in resistance mechanisms, for example *ampD* gene inactivation has been associated to an increased expression of AmpC β-lactamase, which results in an increased β-lactam resistance (Schmidtke and Hanson, 2006), although this has not been shown in *A. baumannii* (Gil-Marqués et al., 2018). In fact, except for fosfomycin, higher susceptibility of Δ*ampD*::Kan and Δ*anmK*::Kan mutant strains to most clinically relevant antibiotics was not observed (Gil-Marqués et al., 2018). Our data showed that deletion of *ampD* and *anmK* also does not affect susceptibility to the disinfectants chlorhexidine and ethanol, the chelating agent EDTA, that disrupts the lipopolysaccharide of the cell wall (Umerska et al., 2018) or the detergent SDS that also acts on the cell wall (Shehadul Islam et al., 2017), and only a small increase in susceptibility to deoxycholate was observed in Δ*ampD*::Kan mutant. This is in line with the idea that resistance to some disinfectants in Gram-negative bacteria is not mediated directly by peptidoglycan, but rather by the presence of efflux pumps in the outer membrane, as previously described (Venter et al., 2015). For example, expression of the multidrug efflux pump AceI has been associated with resistance to chlorhexidine in *A. baumannii* (Bolla et al., 2020). While AcrAB and CmeABC efflux pumps have been associated with resistance to deoxycolate in *Escherichia coli* and *Campylobacter jejuni*, respectively (Thanassi et al., 1997; Lin et al., 2003), for *A. baumannii* it remains to be elucidated if this occurs or it is associated to modifications on lipopolysaccharide. If so, our data points to hypothesise that AmpD may be partly involved, as a restructuring of bacterial envelope may take place. In contrast, the effects of ethanol on bacteria are due to colligative effects instead of damage in a specific receptor, but again mainly affect the integrity of the outer cell membrane (Ingram, 1989; Horinouchi et al., 2018).

Finally, in order to determine how AmpD and AnmK loss affects virulence traits in *A. baumannii*, we characterized biofilm formation and twitching motility, both involved in pathogenesis and transmission of this species (McConnell et al., 2013). *A. baumannii* survival on surfaces is enhanced due to its ability to form biofilms, contributing to its persistence in the hospital environment and increasing the chance of producing infections in the hospital setting (Lin et al., 2020). For the first time, we demonstrate that absence of either AmpD or AnmK results in a tendency towards decreased biofilm formation compared to the wild type strain. The defect on adherence and biofilm formation when peptidoglycan remodelling is altered could be due to alterations in large macromolecular structures needed for biofilm formation, such as pili or protein secretion systems. Those must pass through the peptidoglycan layer of the cell wall to be correctly displayed on the surface of the cell, and often have a large size that typically exceeds the mesh size of peptidoglycan. Therefore, to allow their transit, localized remodelling is required (Gallant et al., 2005). In fact, cell wall remodelling has been shown to be relevant for the assembly of flagella and for type III and type VI secretion systems (Pérez-Gallego et al., 2016). This could also explain the reduced twitching motility in Δ*anmK*::Kan and Δ*ampD*::Kan mutants observed in this work, since type IV pili are necessary for this surface movement (Wall and Kaiser, 1999; Piepenbrink et al., 2016). On the other hand, released peptidoglycan fragments can act as signalling molecules that could also affect motility and biofilm formation (Vermassen et al., 2019; Irazoki et al., 2019). Furthermore, studies carried out in *P. aeruginosa* linked twitching motility to formation and maintenance of biofilm (O'Toole and Kolter, 1998; Chiang and Burrows, 2003), so both virulence traits are related. Although little is known, this relationship has also been observed in *A. baumannii* (Luo et al., 2015), which could explain the defects observed in both virulence traits in the mutant strains used in this study. In addition, biofilm formation has also been related with desiccation tolerance in *A. baumannii* (Espinal et al., 2012) which may facilitate the survival of bacteria in a hospital setting, so it would be of interest to perform further assays to determine if the lack of AmpD and AnmK, also has an effect on resistance to desiccation.

## 5 Conclusion

In this study we demonstrate that the enzymes AmpD and AnmK, both involved in the peptidoglycan recycling pathway, go beyond intrinsic fosfomycin resistance in *A. baumannii*. Different traits related to fitness and virulence are affected when these enzymes are absent, especially AmpD. However, most of the molecular mechanisms that produce these phenotypic changes are still unknown and further analysis are needed to corroborate that these results occur *in vivo*. The findings presented here could be useful for the development of new strategies to fight *A. baumannii* infections. For example, *anmK* and especially *ampD* inhibition could be used in combination with fosfomycin to treat *A. baumannii* infections. In addition, these findings establish a link between fosfomycin resistance and bacterial fitness/virulence in *A. baumannii*.

## Data availability statement

The raw data supporting the conclusions of this article will be made available by the authors upon request, without undue reservation.

## Author contributions

AT: Performed growth curves, MIC, competition, biofilm formation, cell permeability and twitching studies. Analyzed data, drafted the manuscript and revised the final version. MT: Performed microscopy analyses and revised the final version of the manuscript. ML-S: Assisted in experimental procedures, supervised the study, drafted the manuscript and revised the final version. MM: Conceived and supervised the study, revised the manuscript and granted funding. All authors contributed to the article and approved the submitted version.
