# Supplementary material for: Role of peptidoglycan recycling enzymes AmpD and AnmK in Acinetobacter baumannii virulence features
Source: Front Cell Infect Microbiol. 2023 Jan 13;12:1064053. doi: 10.3389/fcimb.2022.1064053 (PMC9880065; doi:10.3389/fcimb.2022.1064053)
Supplement: Supplementary file 1 [file Image_1.pdf]

frontiers

Supplementary Material

Role of peptidoglycan recycling enzymes AmpD and AnmK in *Acinetobacter baumannii* virulence features

Ana Tajuelo[1,2], María C. Terrón[3], Mireia López-Siles[1, 4*#], Michael J McConnell[1#]

[1] Intrahospital Infections Laboratory, National Centre for Microbiology, Instituto de Salud Carlos III (ISCIII), Madrid, Spain

[2] Universidad Nacional de Educación a Distancia (UNED), Madrid, Spain

[3] Electron and Confocal Microscopy Unit, Scientific-Technical Central Units, Instituto de Salud Carlos III (ISCIII), Madrid, Spain

[4] Serra Húnter Fellow. Microbiology of Intestinal Diseases, Biology Department, Universitat de Girona, Girona, Spain

[#] ML-S and MJM contributed equally to this work and should be considered co-senior authors.

*** Correspondence:**
Mireia López Siles
mireia.lopezs@udg.edu

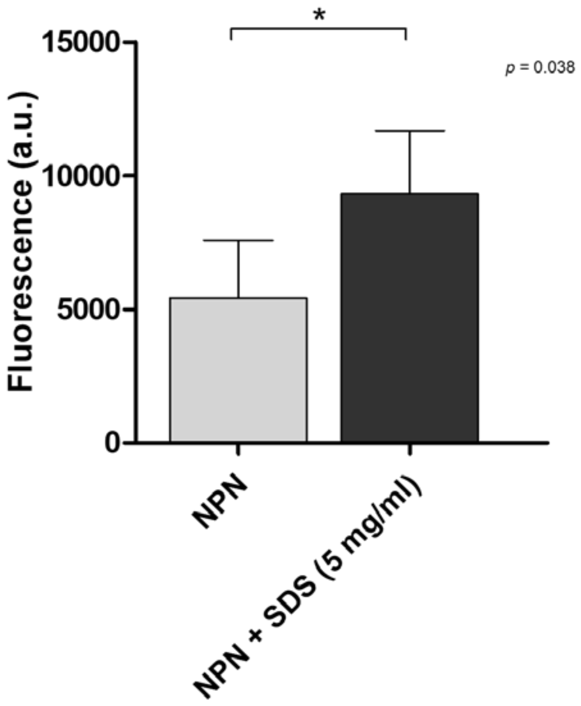

Supplementary Figure 1. Cell permeability of *A. baumannii* ATCC 17978 in the presence of SDS. Effect of detergent SDS on the membrane permeability of *A. baumannii* ATCC 17978 as measured by the 1-N-phenylnaphthylamine (NPN) uptake assay. Bars represent the average of three separate assays, with error bars representing the standard deviation. Significant differences were found between replicates, as assessed by Student's t test.

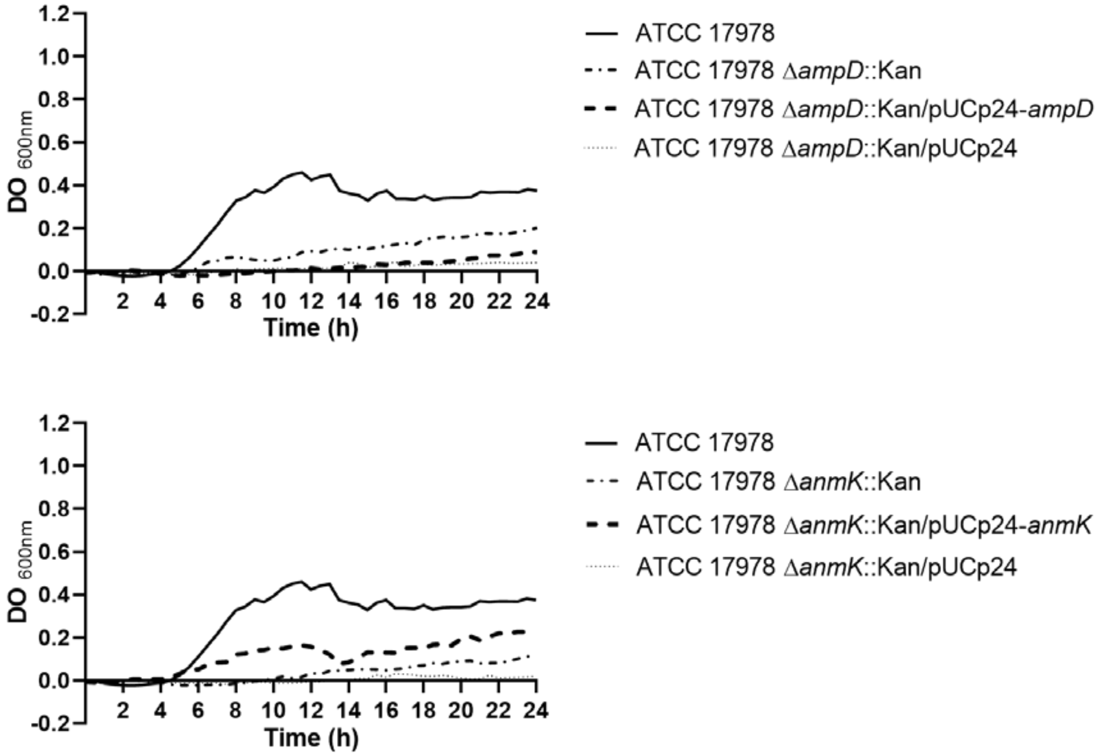

Supplementary Figure 2. In vitro growth of *A. baumannii* strains in serum. Growth curves over 24 h of *A. baumannii* strains in human serum.
